# P-440. Epidemiologic Shift in Pediatric Staphylococcal Scalded Skin Syndrome During the COVID-19 Era: Clinical Predictors and Hospitalization Trends

**DOI:** 10.1093/ofid/ofaf695.655

**Published:** 2026-01-11

**Authors:** Nina K B Gust, Ashley Frei, Rebecca M Adams, Elika Ridelman, Christina Shanti, Ronald Thomas, Jocelyn Y Ang

**Affiliations:** Children's Hospital of Michigan, Royal Oak, MI; Wayne State University School of Medicine, Detroit, Michigan; Wayne State University School of Medicine and Children's Hospital of Michigan, Detroit, Michigan; Wayne State University, Detroit, Michigan; Children's Hospital of Michigan Burn Center, Bloomfield Hills, Michigan; Central Michigan University, Detroit, Michigan; Wayne State University School of Medicine, Detroit, Michigan

## Abstract

**Background:**

Staphylococcal Scalded Skin Syndrome (SSSS) is a blistering skin disorder caused by exfoliative toxins produced by *S. aureus*. The incidence of SSSS in children is ∼7.7 per million, with a mortality rate < 4%. However, comprehensive epidemiologic studies on pediatric SSSS in the United States remain limited.

This study aimed to characterize the demographics, clinical features, and outcomes of children diagnosed with SSSS at an inner-city tertiary care hospital. We also evaluated the impact of the COVID-19 pandemic on incidence and identified clinical factors associated with hospital length of stay (LOS).

**Figure 1:**
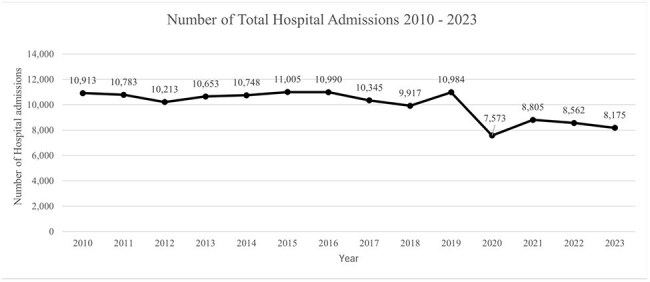
Annual pediatric hospital admissions for all diagnoses from 2010 to 2023

**Figure 2:**
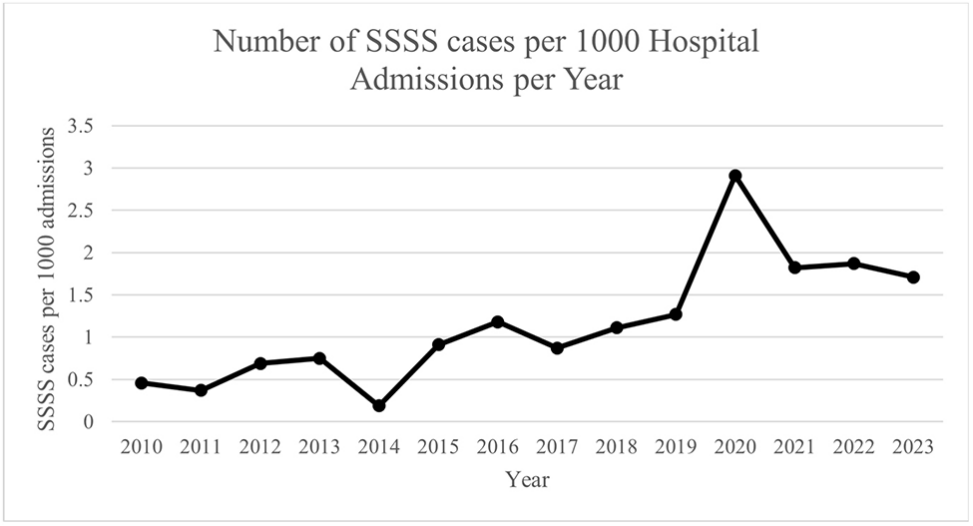
The annual incidence of SSSS per 1,000 hospital admissions (2010 – 2023)

**Methods:**

A retrospective chart review was conducted for patients < 18 years old admitted with a diagnosis of SSSS between 2010 and 2023. Cases were identified using medical records and ICD codes. Data collected included demographic characteristics, clinical presentation, and outcomes. The study period was divided into pre-COVID (2010–2019) and COVID-era (2020–2023) cohorts.

**Table 1:**
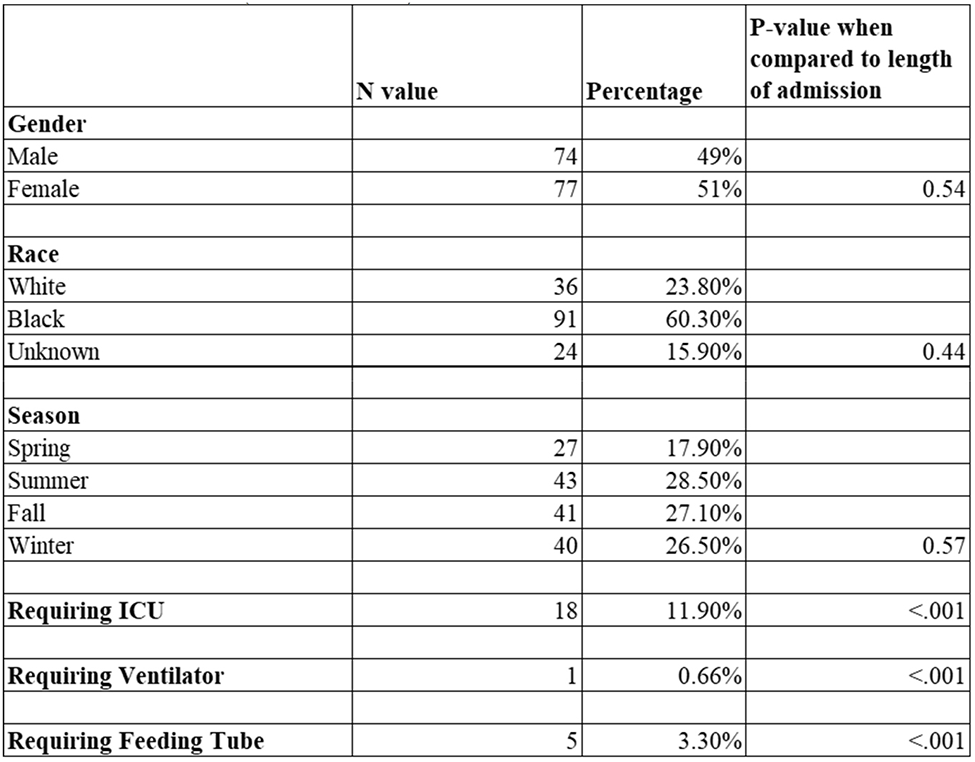
Demographic, seasonal, and clinical factors associated with length of hospital stay in pediatric SSSS patients

**Results:**

A total of 151 patients (ages 0–17, mean 2.4 ± 3.2 years) were included; 51% were female. The average LOS was 3.5 ± 1.9 days. No mortality was observed.

Although total admissions declined significantly during the pandemic (p < 0.001, Figure 1), the annual incidence of SSSS per 1,000 admissions increased. The rise in SSSS incidence during the COVID era compared to the pre-COVID period was statistically significant (p < 0.001, Figure 2).

Demographic and seasonal variables did not differ significantly between the two cohorts. ICU admission, ventilator use, and feeding tube requirement were significantly associated with longer LOS (p < 0.001, Table 1).

**Conclusion:**

Our study did not identify younger age as a predictor of prolonged hospitalization. Instead, higher acuity of illness as indicated by ICU care, mechanical ventilation, and feeding support was associated with increased LOS. We also observed a significant rise in pediatric SSSS incidence during and after the COVID-19 pandemic. This trend may reflect behavioral and societal disruptions in hygiene practices, leading to increased colonization and transmission, as *S. aureus* is primarily spread via close contact. These findings underscore the importance of continued surveillance and early recognition of SSSS in the post-pandemic clinical setting.

**Disclosures:**

Jocelyn Y. Ang, MD, astellas: Honoraria|astellas: speaker bureau|Eli Lilly and Company: Grant/Research Support|F. Hoffmann-La Roche Ltd: Grant/Research Support|Pfizer, Inc.: Honoraria

